# Metabolic Fingerprinting of Murine L929 Fibroblasts as a Cell-Based Tumour Suppressor Model System for Methionine Restriction

**DOI:** 10.3390/ijms22063039

**Published:** 2021-03-16

**Authors:** Werner Schmitz, Corinna Koderer, Mohamed El-Mesery, Sebastian Gubik, Rene Sampers, Anton Straub, Alexander Christian Kübler, Axel Seher

**Affiliations:** 1 Department of Biochemistry and Molecular Biology, Biocenter, D-97074 Wuerzburg, Germany; wschmitz@biozentrum.uni-wuerzburg.de; 2 Department of Oral and Maxillofacial Plastic Surgery, University Hospital Wuezburg, D-97070 Wuerzburg, Germany; corinna.koderer@posteo.de (C.K.); sebastiangubik@web.de (S.G.); r.sampers@web.de (R.S.); straub_a@ukw.de (A.S.); kuebler_A@ukw.de (A.C.K.); 3 Department of Biochemistry, Faculty of Pharmacy, Mansoura University, Mansoura 35516, Egypt; elmesery@hotmail.com

**Keywords:** methionine restriction, caloric restriction, mass spectrometry, LC/MS, liquid chromatography/mass spectrometry, metabolism, L929, amino acid

## Abstract

Since Otto Warburg reported in 1924 that cancer cells address their increased energy requirement through a massive intake of glucose, the cellular energy level has offered a therapeutic anticancer strategy. Methionine restriction (MetR) is one of the most effective approaches for inducing low-energy metabolism (LEM) due to the central position in metabolism of this amino acid. However, no simple in vitro system for the rapid analysis of MetR is currently available, and this study establishes the murine cell line L929 as such a model system. L929 cells react rapidly and efficiently to MetR, and the analysis of more than 150 different metabolites belonging to different classes (amino acids, urea and tricarboxylic acid cycle (TCA) cycles, carbohydrates, etc.) by liquid chromatography/mass spectrometry (LC/MS) defines a metabolic fingerprint and enables the identification of specific metabolites representing normal or MetR conditions. The system facilitates the rapid and efficient testing of potential cancer therapeutic metabolic targets. To date, MS studies of MetR have been performed using organisms and yeast, and the current LC/MS analysis of the intra- and extracellular metabolites in the murine cell line L929 over a period of 5 days thus provides new insights into the effects of MetR at the cellular metabolic level.

## 1. Introduction

Cancer survival has steadily improved in recent decades, but the 5-year survival rates for different types of cancer show marked differences. Specifically, the 5-year survival rate of women in the USA diagnosed with breast cancer during 2010–2014 is over 90%, whereas that of patients with lung cancer in North America is substantially lower than 20% [1,2]. The treatment or even curing of cancer remains an enormous challenge to modern medicine.

The causes of cancer are diverse, and the therapeutic strategies used are also varied. To date, no drug-based therapy has been successfully used for any type of cancer, but there is currently an increasing tendency towards cancer-specific or patient-specific therapy [3,4]. Almost all types of cancer have one feature in common, an increased and accelerated metabolism, which manifests as an increase in the absolute energy requirement. As early as 1924, Otto Warburg and colleagues reported that tumour cells address their increased energy requirements through a massive intake of glucose, which is converted to lactate via “aerobic glycolysis” (Warburg effect) [5]. At that time, it was clear that the increased energy requirement of a tumour offers a possible therapeutic strategy.

The strong importance of the energy level for health and cancer can be observed through a 10–30% reduction in the energy consumed through food—in short, caloric restriction (CR). This approach is very beneficial to health and also extends the life span. CR can thus markedly reduce cardiovascular system-related diseases, type II diabetes and the development of cancer. Astonishingly, these effects have been observed in numerous species, including yeast, *Caenorhabditis elegans*, *Drosophila*, chickens, rats, mice, rhesus monkeys and humans [6]. CR induces complex mechanisms, and the decisive factor is the induction of low-energy metabolism (LEM) mainly through the following four mechanisms: (1) CR leads to a reduction in the growth factor insulin-like-growth-factor-1 (IGF-1) accompanied by an increase in its opponent, fibroblast-growth-factor-21 (FGF-21) [7]. (2) CR reduces/inhibits the activity of mechanistic-target-of-rapamycin (mTOR), which leads to reductions in growth and proliferation [8]. CR (3) strongly induces autophagy [9] and (4) reduces the activity of basic inflammatory mediators [10].

The restriction of carbohydrates or lipids constitutes the first form of caloric restriction but restricting the total amount of amino acids/proteins consumed exerts more fundamental effects, even without a reduction in the total caloric intake [6]. As early as 1959, Sugimura and colleagues showed that a diet lacking an essential amino acid leads to LEM, as demonstrated by the significant weight loss of the test animals [11]. The restriction of one amino acid type also exerts the same effects on health and lifespan as those obtained with CR, and these effects are manifested in the form of an improved glucose metabolism (insulin independent), a reduced tendency toward obesity, marked reductions in cellular proliferation, growth and development and substantially reduced oxidative stress activities at the cellular level [12]. Despite the complexity of the biochemical and metabolic processes associated with the induction of LEM, the effects can be explained at the molecular level. Amino acids form the basis for life in three ways. First, amino acid derivatives constitute some of the main classes of biochemical substances, such as carbohydrates and DNA. Second, amino acid metabolites are involved in all important metabolic pathways, such as TCA and urea cycle. Third, in contrast to carbohydrates and lipids, amino acids do not primarily serve as energy sources but rather play roles as building blocks for structural proteins and enzymes, and these components thus mediate numerous biological functions.

One of the most convincing papers on the effects of protein/amino acid restriction as an anti-tumour strategy was published in 2014 by Levine and colleagues in *Cell Metabolism*. C57BL/6 mice were fed high (18%) or low (4–7%) amounts of protein (measured by the total caloric count) for a period of 39 days, and each of the animals was then subcutaneously implanted with 20,000 murine melanoma cells (B16). After 22 days, the tumour size of the group fed the low amount of protein was significantly smaller than that of the group fed the high protein amount, and on day 39, the tumours of the group fed the high protein amount were on average 78% larger than those of the group fed the low amount of protein. In another experiment, the murine breast cancer cell line 4T1 was tested in BALB7c mice, and the results obtained 18 days after implantation showed that 100% of the control animals developed tumours, whereas only 70% of the animals fed the low protein amount developed tumours. After 39 days, which was the end of the experiment, 20% of the animals in the group fed the low protein amount had still not developed tumours, even though 20,000 living and highly aggressive tumour cells had been implanted. These in vivo experiments show the great potential of protein restriction in cancer therapy [13].

MetR has been established as a type of amino acid restriction due to its central position, which is superior to that of other amino acids. (1) Due to the individual reaction steps required for its synthesis, methionine is the most energy-consuming amino acid and thus a very valuable energy resource [14]. (2) For this reason, methionine has likely phylogenetically established itself as the starting amino acid in almost all organisms. A correspondingly large pool of methionine provides a signal to cell that sufficient energy has been absorbed/is available and that protein synthesis is therefore possible [15]. (3) The *S*-atom of the sulfhydryl group of methionine is essential in sulphur-based metabolic processes, and one example is the synthesis of cysteine from serine. (4) Methionine also plays a central role in many synthetic steps. *S*-adenosylmethionine (SAM) is the key metabolite of the SAM cycle and is formed by methionine-adenosyl transferase with methionine and adenosine triphosphate (ATP) as substrates. Methyl transferases utilize SAM as a methyl donor for a demethylation reaction that yield *S*-adenosyl-homocysteine, which is hydrolysed to adenosine and homocysteine. Homocysteine can be remethylated to methionine or degraded to the amino acid cysteine [16]. The re-methylation of homocysteine is essential for tetrahydrofolate metabolism and consequently for the biosynthesis of purines and pyrimidines.

Relevant studies have shown that MetR is effective against cancer both in vitro and in vivo. Clinical studies have also proven the effectiveness of MetR. In addition to the effect of LEM, numerous tumours are dependent on methionine. Although methionine is essential per se, individual body cells can regenerate the required methionine via different metabolic pathways, e.g., from SAM or homocysteine. Tumours have often lost this ability and are therefore dependent on an external (extracellular) supply of methionine, which describes another advantage of MetR as a therapeutic approach [17,18].

However, no simple in vitro system that can be used for the rapid analysis of MetR and potential therapeutic agents is currently available. One example are the so-called caloric restriction mimetics (CRMs), which are molecules that can induce LEM and thus serve as novel and promising approaches for tumour therapy [19]. We aimed to develop a cell-based in vitro system that enables the rapid and efficient analysis of various factors under MetR conditions. The model system should meet the following requirements. (1) In contrast to human cells, the system should consider the increased metabolism of rodents and react as quickly as possible to MetR with regard to proliferation. Much of the available scientific knowledge is based on the results from studies in mice and rats. (2) The system should react to different factors/ligands and enable a fast read-out. In addition, the factors should exhibit different efficiencies compared with MetR. (3) The metabolism of different metabolic pathways should be important for the definition of a metabolic fingerprint, and the differences in molecule concentrations that specifically define the metabolic profile under MetR and/or LEM could be used to define the potential of a CRM as an energy modulator and its therapeutic potential in cancer.

In this study, we established the murine cell line L929 as a model system. These cells react rapidly and efficiently to MetR. The ligands of the TNFα family, including TNFα and FasL, show significantly different reactivities under normal and methionine-free conditions. The analysis of metabolism over a period of approximately 120 h through the identification of more than 150 molecules belonging to different classes, such as amino acids, amino acid derivates, urea and TCA cycles, carbohydrates, purines, pyrimidines and cofactors, by LC/MS allows the definition of a metabolic fingerprint for these systems and the identification of specific molecules that represent normal or MetR/LEM conditions. As a result, the system facilitates the rapid testing of potential CRMs or cancer therapeutic targets.

## 2. Results

### 2.1. Methionine Restriction Inhibits Effective L929 Cell Proliferation

**Figure 1:**
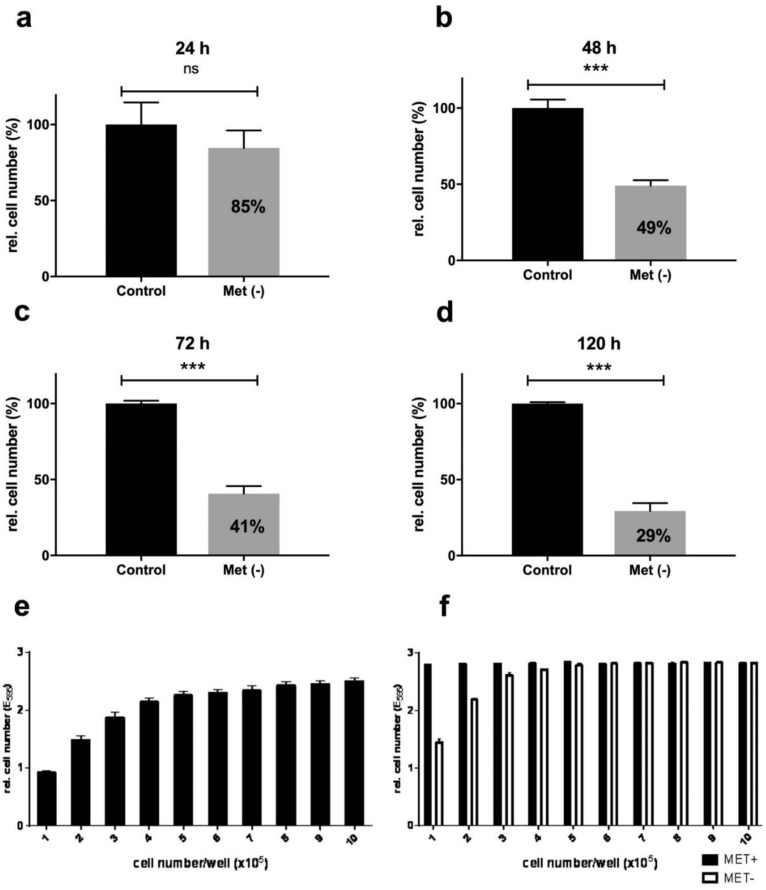
(**a**–**f**) Analysis of L929 cell proliferation in the presence and absence of methionine. A total of 10,000 cells were seeded per well, incubated overnight and stimulated for 24 h, 48 h, 72 h and 120 h with or without methionine. The proliferation of the cells was analysed by crystal violet staining as described in the Materials and Methods. (**a**–**d**) The figures show a summary of the results obtained from four independent experiments (five values for every group). The control was normalized to 100%. (**e**,**f**) Simple analysis of potential cell death. Subfigures (**e**) and (**f)** show the results from one representative experiment. Unpaired Student’s *t*-test was used for comparison between control and Met (*w*/*o*) groups. *p*-values <0.05 indicated statistically significant effects. (ns: non-significant, *** *p* ≤ 0.001).

One of the most prominent effects induced by MetR is the inhibition of proliferation via mTOR [20]. We analysed the inhibitory effect of MetR on the proliferation of L929 cells over a period of 120 h (Figure 1a–d). After 24 h, more than 10% inhibition was observed, whereas more than 50% and 70% inhibition was detected after 48 h and 120 h, respectively. To ensure that the cells did not simply die, we first determined the cell capacity of a well in a 96-well plate. Between 10,000 and 100,000 cells were seeded, and 24 h later, the cells were analysed by crystal violet staining (Figure 1e). The seeding of 50,000 cells per well led to relative confluence (ca. 90%). We then repeated the experiment with a period of 72 h and stimulated the cells with or without methionine (Figure 1f). Starting with 50,000 cells per well, no relevant difference in the relative cell number were found between normal and methionine restricted conditions. The induction of cell death via MetR should result in a significantly reduced cell number compared with that obtained with the control conditions.

### 2.2. MetR Changes TNFα Ligand and Cytostatic Sensitivity in L929 Cells

**Figure 2:**
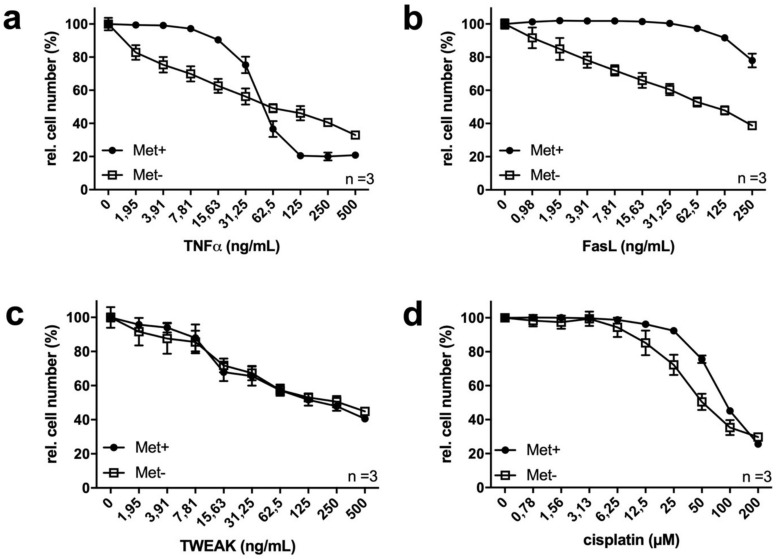
(**a**–**d**) Methionine restriction sensitizes L929 cells to TNFα ligands and cisplatin. A total of 10,000 cells/well were seeded, incubated overnight and then stimulated for 72 h with or without methionine in combination with TNFα (**a**), FasL (**b**), TWEAK (**c**) or cisplatin (**d**). Cell proliferation was analysed by crystal violet staining as described in the Materials and Methods. The figures show a summary of the results from three independent experiments. The control was normalized to 100%.

In a model system, MetR should also influence the sensitivity of different ligands and reagents. For this reason, we tested three different ligands from the tumour necrosis factor alpha (TNFα) family, namely, TNFα, Fas ligand (FasL) and tumor necrosis factor-like weak inducer of apoptosis (TWEAK), as well as cytostatic cisplatin. Both TNFα (Figure 2a) and FasL (Figure 2b) showed increased sensitivity at low concentrations, whereas no significant change in TWEAK was detected (Figure 2c). However, under MetR, saturation curve (as shown for TNFα under complete medium conditions) is not typical but rather linear. For cisplatin, only a slight increase in sensitivity was observed in the absence of methionine (Figure 2d).

### 2.3. MetR Induces Metabolic Reprogramming in L929 Cells

To analyse the influence of MetR on the metabolism of L929 cells, a total of 157 different water-soluble metabolites belonging to the classes of amino acid, amino acid derivatives, urea and TCA cycles, carbohydrates, lipid-intermediates, pyrimidines, purines and cofactors were analysed over a period of 120 h at 24 h intervals by LC/MS. In this analysis, both the intracellular (cell pellets) and extracellular (medium) metabolites were analysed. An overview of all analysed metabolites and results is shown in the supplementary information. Due to the large amount of data, only selected and decisive areas are addressed here; specifically, we focused on the following areas:

(1) important metabolic pathways
(2) metabolites that are directly dependent on methionine
(3) metabolites that are indirectly dependent on methionine
(4) energy currencies (ATP, NADH, etc.)

**Figure 3:**
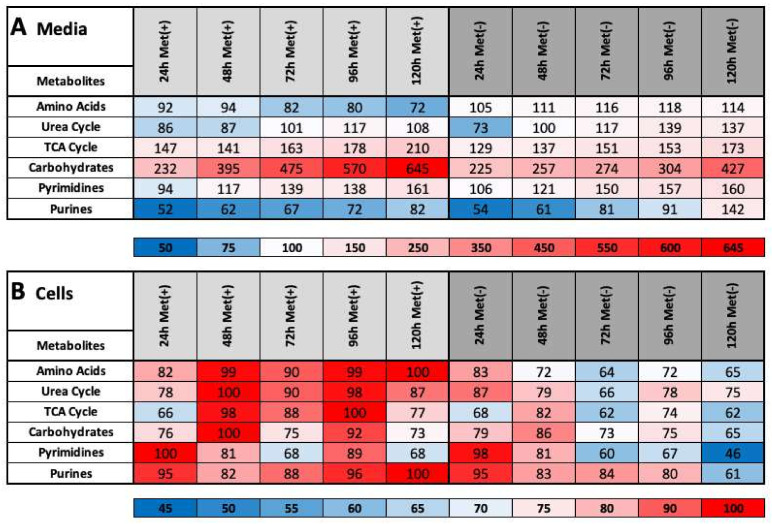
(**A**,**B**) Heat map overview of the summarized metabolites belonging to various classes and associated with various pathways in cell pellets and media. The percentages of the metabolites classified as amino acids, carbohydrates, pyrimidines and purines and belonging to the urea and TCA cycle pathways in media (**A**) and cells (**B**) were calculated as described in the Materials and Methods.

In L929 cells, MetR clearly induces LEM and a constant reprogramming of important metabolic pathways. These findings are clarified by their sum of the metabolite masses associated with these pathways. In addition to the meaningfulness of the relative concentration of individual molecules, the summed relative masses of all relevant metabolites belonging to one pathway (e.g., TCA cycle) provide relevant information about its overall regulation. This type of analysis was performed for amino acids (except methionine), the TCA cycle, the urea cycle, carbohydrates, pyrimidines and purines in both media (Figure 3A) and cell extracts (Figure 3B).

Although most of the trends were similar, the classes “amino acids” and “purines” stand out. Specifically, the total intracellular amounts of amino acids decrease (from 83 to 65) in response to MetR, whereas the total extracellular amount increases (from 105 to 114) due to secretion. The exact opposite result was found for cells incubated with complete medium: the extracellular and intracellular concentrations decreased (from 92 to 72) and increases (from 82 to 100) in response to MetR. Additionally, different results were obtained for purine metabolism. Under MetR, the purine level in media presented a greater increase (from 54 to 142) compared with that found under control conditions, but the intracellular level markedly decreased (from 95 to 61).

**Figure 4:**
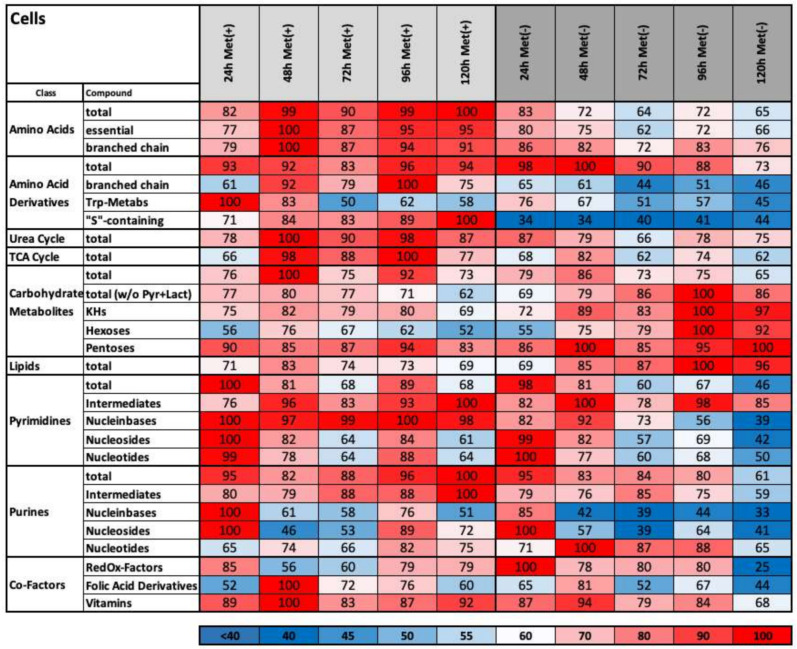
Heat map overview of the summarized metabolites belonging to different pathways in cell pellets. The percentages of the summarized metabolites belonging to the classes of amino acids, amino acid derivates, urea and TCA cycles, carbohydrates, lipids, pyrimidines, purines and cofactors were calculated as described in the Materials and Methods.

**Figure 5:**
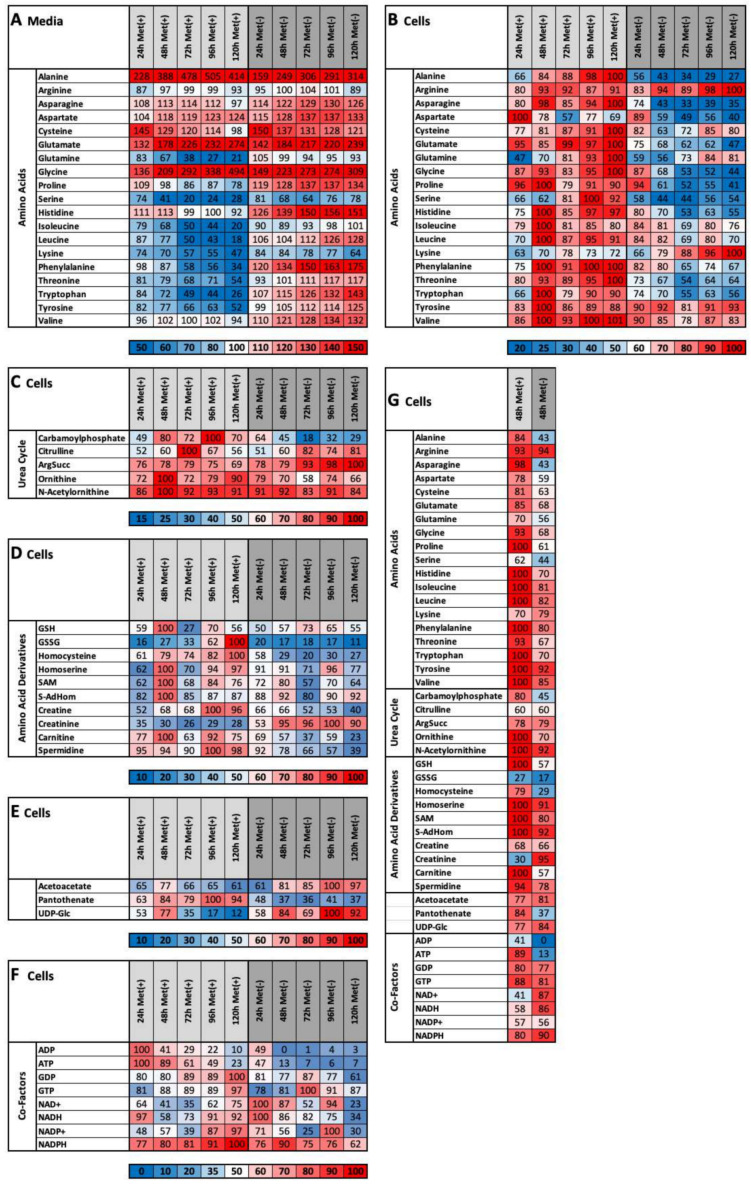
(**A**–**G**) Heat map overview of selected metabolites. The percentages of intracellular (**A**), extracellular (**B**) and nonproteinogenic (urea cycle) (**C**) amino acids, directly-cysteine-dependent (**D**) and indirectly-cysteine-dependent (**E**) amino acids and energy currencies (**F**) were determined as described in the Materials and Methods. (**G**) The results after 48 h that define the candidates for metabolic fingerprint are shown.

The metabolic groups can be divided into further subgroups (Figure 4). The amino acid content decreased under methionine-free conditions for essential (from 80 to 66) and branched types (from 86 to 76). Among the group of amino acid derivates, the “S”-containing group is of most interest. The absence of a sulphur source from methionine decreases the amount of “S”-containing metabolites after 24 h. Amazingly, the amount is maintained between 34% and 44% over a period of 5 days. Thus, the cell places great importance on protecting this valuable resource. Additionally, the different carbohydrate subgroups indicate that the cells under MetR appear to accumulate carbohydrates as an energy source. These results are also reflected by the increase in the amount of intracellular UDP-glucose (Figure 5), the starting molecule in glycogen synthesis. Another marker for LEM is a strong increase in intracellular lipid storage (96%) compared with that found under control conditions (69%). In contrast, nucleotide metabolism for pyrimidines and purines showed a stronger decrease under MetR conditions due to their nucleobases, nucleosides and nucleotides. Another strong indicator of energy metabolism is the RedOx factor, which decreases to 25% at day 5 under MetR conditions.

The effects of MetR become particularly clear at the level of single metabolites (Figure 5A–G). As described previously, the total intracellular amounts of amino acids decreased under MetR. Significant exceptions are the amino acids arginine, lysine and tyrosine and, to a lesser extent, glutamine, valine and cysteine, which are still present at high or higher intracellular concentrations (Figure 5A). The opposite effect can be observed for amino acids at the extracellular level. Metabolites in which methionine (or cysteine) forms part of the synthesis are shown in Figure 5D. The amounts of glutathione disulphide (GSSG), homocysteine, homoserine, *S*-adenosylhomocysteine (*S*-AdHom), creatine, carnitine and spermidine decrease significantly for the first 5 days of MetR. The level of glutathione (GSH) decreases under both methionine-rich and methionine-free conditions, but the GSH content is significantly lower under methionine-free conditions. The exceptions are the two products SAM and creatinine, which exhibit continuous increases under methionine-free conditions. The continuous increases in acetoacetate and UDP-glucose as well as the extremely low level of pantothenic acid under methionine-free conditions are striking because their products are not directly dependent on methionine but show significant differences under MetR (Figure 5E).

LEM is particularly well reflected at the level of energy currencies. A lack of energy (e.g., ATP and ADP) is clearly observed under methionine-free conditions, particularly on day 5. Strikingly, the exact opposite was found for NAD^+^, NADH, NADP^+^ and NADPH. Under methionine-free conditions, the synthesis increases during the first two days and then decreases, whereas the synthesis under complete medium conditions increases continuously during the first 5 days (Figure 5F).

### 2.4. Methionine Restriction Induces a Characteristic Metabolic Fingerprint in L929 Cells

The stated goal of this study was to identify a characteristic metabolic profile of L929 cells, i.e., a metabolic fingerprint that enables the rapid and easy analysis and identification of potential inducers of LEM (e.g., so-called caloric restriction mimetics (CRM)) by simply comparing a new metabolic profile of L929 cells with the fingerprint defined in this study for MetR. Based on the number of matches among the differentially synthesized metabolites, the potential of individual active ingredients can be analysed and assessed. Figure 5G shows a summary of the LC/MS results obtained for intracellular metabolites under MetR for 48 h. The heat map clearly shows that after 48 h, a specific profile—a metabolic fingerprint—can be defined and that many of the metabolites shown are present at very different concentrations at this time point. The fingerprint becomes increasingly specific and accurate over time, as both the difference in the amounts of molecules and the number of metabolites that are differential increase under MetR.

Certain components are suitable individually or in combination for “fast tracking”, a rapid and easy method for determining the induction of a LEM. Although MS is efficient, the analysis takes a certain amount of time and money, and not every laboratory can perform this method at all times. In addition to the large fingerprint with numerous metabolites, the induction of a LEM can potentially be analysed using a small footprint, i.e., selected metabolites and alternative measurement methods (particularly ELISA or enzymatic assays). In our view, the combination of acetoacetate, creatine, spermidine, GSSG and the ratio of ADP/ATP is particularly suitable for determining LEM in L929 cells. Both the intracellular and extracellular levels of all the components increase or decrease specifically over a period of 5 days; thus, these components can be easily obtained and determined through an analysis of the supernatant. Simple commercial kits are available for all components, and acetoacetate and creatinine can also often be determined inexpensively via clinical chemistry. Other factors that can be included for better determination are pantothenic acid, UDP-glucose and the amino acids secreted in the media.

## 3. Discussion

Caloric restriction, particularly MetR, is one of the most effective methods for inducing LEM. However, no simple in vitro system for analysing MetR and potential therapeutic agents, such as caloric restriction mimetics, is currently available. Additionally, scarce MS data are available on the metabolism of MetR for the metabolism for single cell/cell lines. In this study, we establish the murine cell line L929 as a model system by analysing the metabolic profile of these cells under MetR by LC/MS and define a “metabolic fingerprint” and a smaller so-called “metabolic footprint”.

The proliferation behavior of the murine cell line L929 was tested over approximately 5 days. L929 cells react rapidly and efficiently to MetR. After 48 h, 50% inhibition of proliferation was observed, and this inhibition decreased to 30% after 120 h. To exclude the possibility that the cells did not simply die as a result of malnutrition, we analysed L929 cells under confluent conditions (Figure 1e,f). After 72 h, no significant reduction in the cell number was found compared with the control. In further experiments, we also analysed the optical viability of the cells via microscopy and additional viability assays (WST-1). In all cases, the cells were viable, and no optical or metabolic hint of significant cell death was detected.

Interestingly, TNFα and FasL showed significantly different reactivities under normal and MetR conditions. The sensitivities of both ligands were increased at low concentrations, but the responsivity of TNFα changed at higher concentrations. The increase in sensitivity is likely due to several factors because the function corresponds more to a straight line than a saturation curve. This is the case if the measured activity does not correspond to a direct 1:1 interaction (e.g., ligand and receptor binding), which indicates that it becomes saturated with increasing concentration but involves several reactions. MetR can influence the expression level of different genes [9] and activate or inhibit different signal transduction pathways, such as mTOR or AMP-kinase [21]. In total, the cells do not react under saturation conditions but rather in a step-by-step (linear) manner when several factors are required to increase the activity or responsiveness.

The investigation of metabolism via a LC/MS analysis of more than 150 different water-soluble metabolites belonging to the classes amino acids, amino acid derivates, urea and TCA cycles, carbohydrates, purines, pyrimidines, lipid intermediates and cofactors defines a metabolic fingerprint of the system and identifies specific molecules that represent MetR (LEM) conditions. To date, MS studies of MetR have mainly been performed in organisms such as mice (e.g., [22,23]). The analysis of the intra- and extracellular metabolites in a murine cell line over a period of 5 days by LC/MS is thus a novelty.

The metabolic profile clearly shows the implementation of LEM. One of the most prominent effects is a decreased intracellular amount of amino acids, which is due to a lack of uptake accompanied by increased secretion of amino acids under MetR. This finding appears to be characteristic under MetR conditions for some important metabolites (e.g., spermidine, creatine, creatinine, choline, homocysteine, and NADPH). There is a low or high intracellular concentration and a high or low extracellular concentration, respectively, in complete medium. We postulate that these molecules are excellent markers for MetR and optional for LEM. However, what are the reasons for this type of reaction? Amino acids are valuable resources, particularly under low-energy conditions. There are two main reasons for this finding: the energy situation and cell communication. The body and thus different tissues are units with specialized functions based on the principle of division of work, but every cell works in a system based on altruistic rules for the whole biological system—the organism. The secretion of amino acids under MetR or LEM is a possible altruistic reaction. Amino acids that are not used will be secreted and thus serve as nutrients for other cells, e.g., the liver synthesizes glucose via gluconeogenesis from keto acids (such as oxaloacetate) generated from amino acids. Another example is acetoacetate, which is related to a low energy level and induces a switch to lipid metabolism. This energy-rich molecule is secreted by L929 cells under MetR.

The secreted molecules could also serve as signal molecules. One excellent candidate for this role is spermidine. Spermidine plays a special role in LEM. Therefore, the amount of spermidine serves as a transmitter for the energetic state of a cell. If the amount of spermidine in a cell decreases, as in L929 cells under MetR, protein synthesis is markedly reduced, and cell proliferation is inhibited [24]. Spermidine, as a polyamine, interacts with a variety of molecules within a cell, including ribosomes. The binding of spermidine promotes protein synthesis, whereas its elimination inhibits the synthesis of proteins. Spermidine is thus one of the main sensors alongside mTOR for methionine [20,25] because decarboxylated SAM is required for its synthesis from putrescine. This finding could also be related to the increased secretion of spermidine from L929 cells, which provides a signal to the surrounding cells of an energy deficit. The uptake of extracellular spermidine can increase the autophagy of cells, which is another characteristic process of cells under LEM [26]. However, the difference between intracellular and extracellular spermidine is unclear because the absorbed spermidine actually increases the intracellular level. Spermidine might be modified during uptake or directly linked to autophagy via specific vesicle transport or is metabolically converted.

LEM is also strong reflected at the energy unit level. After 5 days, ADP, ATP, GDP, NAD^+^, NADH, NADP^+^ and NADPH show a marked decrease compared with the levels found under control conditions. These precise molecules are crucial for the initiation of LEM. The ADP/ATP ratio is controlled by the AMP-kinase, which can inhibit mTOR and thus cellular proliferation and growth [19,27]. NAD^+^ is also a strong inducer of LEM and can also act on AMP-kinase and thus mTOR via sirtuins, a group of histone deacetylases [28,29].

The effects of MetR on metabolites whose synthesis is directly dependent on methionine/cysteine are also clearly recognizable. GSSG, homocysteine, homoserine, S-adenosylhomocysteine, SAM, creatine, creatinine, carnitine and spermidine are regulated by very differential processes. However, despite a lack of methionine, the synthesis does not consistently decrease with decreases in all metabolites. Strikingly, the contents of both S-adenosylmethionine and creatinine exhibit marked increases. An increase in SAM could indicate the general storage of more or less methionine. SAM is so important that it can be converted into various metabolic forms, including methionine [17]. The presence of creatinine might indicate an increased recovery of ATP from oxidative phosphorylation. Creatine phosphate primarily serves as an energy buffer in muscle and can regenerate ADP back to ATP in cases when energy is rapidly needed. In the recovery phase, creatine is primarily re-phosphorylated via a membrane-borne mitochondrial creatine kinase. Creatinine is the product of the degradation of creatine. An increasing content of creatinine could indicate a higher need/conversion of creatine and a shift in ATP production via oxidative phosphorylation [30].

As mentioned previously, a continuous decrease in the intracellular level of amino acids is observed under MetR. Arginine, lysine and, to a lesser extent, glutamine, valine and tyrosine were excluded. These amino acids show marked increases (or no decrease) and appear to play an important role in MetR. First, arginine plays a central role and is involved in NO synthesis, the synthesis of ornithine (urea cycle) and creatinine and in the synthesis of the polyamine spermidine, which is markedly reduced in L929 cells under MetR [25,31,32]. Lysine is an essential and ketogenic amino acid. Together with methionine, lysine is responsible for the formation of l-carnitine, which is essential for the transport of fat in the mitochondria—with the branched-chain amino acids (BCAA = leucine, isoleucine and valine), lysine is an excellent source of acetyl-CoA. With the exception of serving as a pure energy source that generates NADH and FADH_2_ via the TCA cycle, acetyl-CoA plays a critical role in protein acetylation processes similar to histones [25]. As a result, lysine might play a role as an acetyl-CoA energy source under MetR. Glutamine is generally a central amino acid in metabolism. Glutamine is mainly anaplerotic, and both amine groups support the synthesis of TCA metabolites. In cancer cells, glutamine generates α-ketoglutarate and oxaloacetate for TCA metabolism through a biochemical process called glutaminolysis [26]. Together with glycine and aspartate, glutamine serves as a C-atom and nitrogen donor for purine biosynthesis [33]. Another important biological pathway is the so-called “nonessential amino acid synthesis” (NEAAS). Through this process, glutamine allows the synthesis of different amino acids and could serve as a NEAA pool in L929 cells under MetR. One option is the conversion of glutamine into glutamate through a reaction catalysed by the enzyme glutaminase. Glutamate can then be further converted to alanine, aspartate or phosphoserine [31]. Tyrosine is the starting substance for the synthesis of a number of other metabolites, such as DOPA, dopamine, catecholamines, melanin, thyroxine and tyramine. Whether this is the primary cause of storage remains the subject of further investigation.

Two other general metabolic pathways are of interest in the context of MetR, which will only be discussed briefly here—lipid oxidation and glycolysis. MetR differs from a state of hunger insofar as the metabolism does not generally switch to the synthesis of ketone bodies but promote the catabolism of lipids. Selected metabolites such as ketone bodies, carnitine, lactate and CoA are good indicators. In our analysis, the acetoacetate and hydroxybutyrate of the group of ketone bodies were measured. While acetoacetate can also be formed as an intermediate product in general oxidation of fat and amino acids, hydroxybutyrate is mainly formed in glucose starving cells for longer time. The cells under MetR show a steadily increasing level of acetoacetate as evidence of increasing lipid catabolism, but the content of hydroxybutyrate remains more or less the same. MetR differs significantly from the starvation metabolism. The CoA content is almost the same under both conditions. Carnitine, which is required for lipid transport is also reduced under MetR. Lactate is also a good metabolic indicator and plays a key role in the aforementioned Warburg effect. Under MetR conditions lactate production is lower within the first 48 h compared to control. This can be an indication of a decrease in glycolysis, or an indication of decreasing proliferation. An increased lactate production is not a tumour-specific phenomenon but in general for proliferating cells [32].

It is noticeable that within the first 72 h intermediate products of glycolysis accumulate more in the cells under Met(+) conditions (e.g., hexose phosphate, fructose-1,6-bisphosphate, dihydroxyacetone phosphate, 3-phosphoglycerate). This effect changes after 72 h. Glycolysis metabolites are now accumulated under Met(−) conditions (3-phosphoglycerates, phosphoenolpyruvates, pyruvates). However, glycolysis is the most difficult metabolic pathway to evaluate. Regulatory interventions in central glycolysis enzymes such as hexokinase or pyruvate kinase can lead to a massive shift in the steady state within seconds. Further and specialized analyzes are necessary here with isotope-labeled metabolites (glucose), which then allow a much more precise interpretation (the data mentioned are listed in the Supplementary Materials).

In summary, MS analysis allows the definition of a metabolic fingerprint that enables the identification of a MetR or low-energy specific profile. As shown in Figure 5G, many of the selected metabolites show significant differences after 48 h, and this finding enables the analysis of, e.g., so-called caloric restriction mimetics, which are generally able to induce LEM. Some examples are rapamycin, NAD^+^, resveratrol and metformin [19]. However, not every laboratory is able to perform MS analysis. From our point of view, the “small footprint”, the combination of spermidine, creatine, acetoacetate, GSSG and the ratio of ADP/ATP allows a simple “fast tracking” analysis using commercially available kits for the analysis of LEM. As a result, the system facilitates the rapid and efficient testing of potential CRMs or cancer therapeutic metabolic targets.

The analysis of metabolism under MetR also allows the identification of potential targets for cancer therapies. One of the main hypotheses regarding MetR or CR is that the reduced energy level leads normal cells to a situation in which growth and proliferation are markedly reduced, autophagy is induced, and intracellular energy is used to stabilize the genome via epigenetic factors (sirtuins), which would prevent the ageing processes that ultimately promote cancer [19,29,34,35]. In contrast, cancer cells do not exhibit a low energy level and are programmed to grow and divide under most cellular conditions by ignoring the majority of extrinsic and intrinsic signals. The enormous ego of cancer cells allows a great opportunity to attack these cells. Our results show that many amino acids, with the exception for example of arginine or glutamine, are secreted. Amino acid metabolism and thus the inhibition of membrane-based amino acid transporters have become highly relevant over the last decade [36]. What happens when MetR and a glutamine transporter inhibitor are combined in cancer therapy? Glutamine has great relevance for tumour cells, particularly for their growth [26]. In our experiment, L929 cells generate a glutamine pool. The cells appear to not be dependent on extracellular glutamine, and their proliferation is inhibited or strongly reduced. As a result, a glutamine transport inhibitor can potentially hit the tumour but not the somatic cells in LEM. As an example, the pharmacological blockade of the glutamine amino acid transporter ASCT2 using the inhibitor V-9302 results in attenuated cancer cell growth and proliferation, enhanced cell death, and increased oxidative stress, and these effects collectively contribute to anti-tumour responses in vitro and in vivo [37].

In the future, we need more metabolic data from more cell types, particularly cancer cells, for the analysis of LEM under MetR and thus the identification of specific targets that are useful in cancer therapy.

## 4. Materials and Methods

### 4.1. Cell Culture

The murine fibroblast cell line L929 was purchased from Leibniz Institute, DSMZ-German Collection of Microorganisms and Cell Cultures GmbH (Braunschweig, Germany). The cells were cultured in RPMI medium 1640 without or with 15 mg/L methionine (Gibco, Life Technologies; Darmstadt, Germany) and with 1% penicillin/streptomycin (P/S; 100 U/mL penicillin and 100 µg/mL streptomycin; Thermo Fisher Scientific, Darmstadt, Germany) at 37 °C in a humidified atmosphere containing 5% CO_2_. In general, the normal medium is called control medium or Met(+) medium. The composition of the Met(−) medium corresponds to the control medium without the amino acid methionine.

### 4.2. Recombinant Protein Expression

The plasmids for the expression of TNFα, FasL and TWEAK were provided as a gift by Professor Harald Wajant (Division of Molecular Internal Medicine, Department of Internal Medicine II, University Hospital Würzburg). The proteins were expressed as previously described [34,35,38]. In short, affinity chromatography with anti-FLAG M2 agarose beads (Sigma-Aldrich, Darmstadt, Germany) was performed to purify human recombinant FLAG-tagged soluble Fc-FLAG-FasL, FLAG-TNFα and TWEAK-FLAG from the supernatants of HEK293 cells that were stably transfected with the corresponding expression plasmid. The LPS contents were verified using the Pierce LAL chromogenic endotoxin quantification kit (Thermo Fischer Scientific).

### 4.3. Crystal Violet Staining (CytoTox Assay)

The cells were seeded at a minimal amount of 10,000 cells in 100 µL of culture medium per well of a 96-well plate in triplicate and incubated overnight. The cell number per well is mentioned in the legend of the figures showing the experimental results. The following day, the cells were incubated in complete or methionine-free media with or without ligands (TNFα, FasL or TWEAK) or cisplatin (Merck, Darmstadt, Germany) at the indicated concentration. The incubation time is also mentioned in the corresponding figure legend. For staining, the supernatants were removed, and the cells on each well were incubated with 50 µL crystal violet solution (1% crystal violet in 20% methanol; Carl Roth, Karlsruhe, Germany) for 10 min and subsequently washed five times with distilled water. The plates were dried for 24 h in the dark. For quantification, 100 µL of methanol was added to each well, and the plate was incubated for 10 min until the crystal violet was completely dissolved. The photometric absorbance was measured at 595 nm using a microplate reader (Tecan, Crailsheim, Germany). For data analysis, the experiments were repeated three times to calculate the mean values and standard deviations (*n* = 3). The results were normalized to the untreated control (100%). Therefore, the relative cell number values determined via the crystal violet assay with the stimulated probes (CV**_S_**) were normalized to those of the untreated control (CV**_C_**) ((CV**_S_**/CV**_C_**) = CV**_R_**). To obtain percentage values, the CV**_R_** value was multiplied by 100 (RCN (%) = (CV**_S_**/CV**_C_**) × 100 = CV**_R%_**). For statistical analysis, these results were evaluated with unpaired Student’s *t*-test. The significance level was set to *p* < 0.05.

### 4.4. Liquid Chromatography/Mass Spectrometry

Analysis of water-soluble metabolites in cell extracts and culture media:

#### 4.4.1. Cells

After the addition of 0.5 mL of MeOH/CH_3_CN/H_2_O (50/30/20, *v*/*v*/*v*) containing 10 µM lamivudine, the cell pellets were homogenized by ultrasound treatment (10 × 1 s. 250 W output energy). Media: One hundred µL of culture medium was combined with 0.4 mL of MeOH/CH_3_CN (50/30, *v*/*v*) containing 10 µM lamivudine. General procedure: The resulting suspension was centrifuged (20 kRCF for 2 min in an Eppendorf centrifuge 5424), and the supernatant was applied to a C18-SPE column that was activated with 0.5 mL of CH_3_CN and equilibrated with 0.5 mL of MeOH/CH_3_CN/H_2_O (50/30/20, *v*/*v*/*v*). The SPE eluate was evaporated in a vacuum concentrator. The resulting pellet was dissolved in 50 µL (cell extracts) or 500 µL (media extracts) of 5 mM NH_4_OAc in CH_3_CN (25/75, *v*/*v*).

#### 4.4.2. LC parameters

Mobile phase A consisted of 5 mM NH_4_OAc in CH_3_CN/H_2_O (5/95, *v*/*v*), and mobile phase B consisted of 5 mM NH_4_OAc in CH_3_CN/H_2_O (95/5, *v*/*v*). After application of 3 µL of the sample to the ZIC-HILIC column (at 30 °C), the LC gradient programme was as follows: 100% solvent B for 2 min, linear decrease to 40% solvent B over 16 min, maintenance at 40% solvent B for 9 min, and increase to 100% solvent B over 1 min. The column was maintained at 100% solvent B for 5 min for column equilibration before each injection. The flow rate was maintained at 200 μL/min. The eluent was directed to the ESI source of the QE-MS from 1.85 min to 20.0 min after sample injection.

#### 4.4.3. MS Parameters

Scan type: full MS in the positive-and-negative mode (alternating); scan range, 69–1000 *m*/*z*; resolution, 70,000; AGC-target, 3E6; maximum injection time, 200 ms; sheath gas, 30; auxiliary gas, 10; sweep gas, 3; spray voltage, 3.6 kV (positive mode) or 2.5 kV (negative mode); capillary temperature, 320 °C; S-lens RF level, 55.0; and auxiliary gas heater temperature, 120 °C. Annotation and data evaluation: Peaks corresponding to the calculated monoisotopic masses (MIM ± H^+^ ± 2 mMU) were integrated using TraceFinder software (Thermo Scientific, Bremen, Germany). Materials: Ultrapure water was obtained from a Millipore water purification system (Milli-Q Merck Millipore, Darmstadt, Germany). HPLC–MS solvents, LC–MS NH_4_OAc and lamivudine were purchased from Merck. RP18-SPE Columns: 50 mg Strata C18-E (55 µm) in 1 mL tubes (Phenomenex, Aschaffenburg, Germany). Sonifier: Branson Ultrasonics 250 equipped with a 13 mm-Disintegrator-Sonotrode (Thermo Scientific). LC/MS-system: Thermo Scientific Dionex UltiMate 3000 UHPLC system hyphenated with a Q Exactive mass spectrometer (QE-MS) equipped with a HESI probe (Thermo Scientific). Particle Filter: Javelin Filter with an ID of 2.1 mm (Thermo Scientific). UPLC-precolumn: SeQuant ZIC-HILIC column (5 μm particles, 20 × 2 mm) (Merck). UPLC column: SeQuant ZIC-HILIC column (3.5 μm particles, 100 × 2.1 mm) (Merck).

#### 4.4.4. Raw Data Analysis and Value Generation (In Short):

LC/MS analyses were carried out as published before [39,40] in four independent experiments at 24 h, 48 h, 72 h, 96 h and 120 h, each value in triplicate. Metabolites were quantified in cell pellets and corresponding supernatants (media) under methionine-containing and methionine-free conditions (12 samples per time point in total). The resulting peak areas were normalized against lamivudine as external standard. From this, the mean value was calculated for each triplicate, including the standard deviation. For better comparison, the values have been converted to percent. For the values of the media, the control measurement of the medium used, would be defined as 100%. For the cell pellets, the highest measured value in each test series within an experiment was defined as 100%. From these values, the average mean values from the four experiments were then summarized in the individual tables. For a better overview, the results were rounded to natural numbers and shown as a heat map. The corresponding colour range is indicated individually under each figure/table. All values, their individual calculations and the data sets used for the individual tables is added as a Word file in the Supplementary Materials.

### 4.5. Statistical Analysis

Data collection and plotting were performed with Excel (Microsoft, Redmond, WA, USA) and GraphPad Prism (v. 6.04; GraphPad Software, San Diego, CA, USA) software. The statistical analyses were accomplished with GraphPad Prism and MEDAS (Grund EDV-Systeme, Margetshöchheim, Germany) software. Unpaired Student’s *t*-test was used for comparison between control and Met (w/o) groups. *p*-values <0.05 indicated statistically significant effects. (ns: non-significant, *** *p* ≤ 0.001).

## Supplementary Materials

The following are available online at https://www.mdpi.com/1422-0067/22/6/3039/s1, word document “Total Overview Result LC/MS”.
