# Supplementary material for: Metabolic Fingerprinting of Murine L929 Fibroblasts as a Cell-Based Tumour Suppressor Model System for Methionine Restriction
Source: Int J Mol Sci. 2021 Mar 16;22(6):3039. doi: 10.3390/ijms22063039 (PMC8002350; doi:10.3390/ijms22063039)
Supplement: Supplementary file 1 [file ijms-22-03039-s001.pdf]

Total Overview Result LC/MS

Media

Class	Compound	24h Met(+)	48h Met(+)	72h Met(+)	96h Met(+)	120h Met(+)	24h Met(-)	48h Met(-)	72h Met(-)	96h Met(-)	120h Met(-)
Amino Acids	Alanine	228	388	478	505	414	159	249	306	291	314
	Arginine	87	97	99	99	93	95	100	104	101	89
	Asparagine	108	113	114	112	97	114	122	129	130	126
	Aspartate	104	118	119	123	124	115	128	137	137	133
	Cysteine	145	129	120	114	98	150	137	131	128	121
	Glutamate	132	178	226	232	274	142	184	217	220	239
	Glutamine	83	67	38	27	21	105	99	94	95	93
	Glycine	136	209	292	338	494	149	223	273	274	309
	Proline	109	98	86	87	78	119	128	137	137	134
	Serine	74	41	20	24	28	81	68	64	76	78
	Methionine	86	71	46	41	17	69	38	22	18	14
	Histidine	111	113	99	100	92	126	139	150	156	151
	Isoleucine	79	68	50	44	20	90	89	93	98	101
	Leucine	87	77	50	43	18	106	104	112	126	128
	Lysine	74	70	57	55	47	84	84	78	77	64
	Phenylalanine	98	87	58	56	34	120	134	150	163	175
	Threonine	81	79	68	71	54	93	101	111	117	117
	Tryptophan	84	72	49	44	26	107	115	126	132	143
	Tyrosine	82	77	66	63	52	99	105	112	114	125
	Valine	96	102	100	102	94	110	121	128	134	132
Amino Acid Derivatives	Cystine	654	265	46	53	0	######	######	######	######	######
	GSH	253	337	519	227	200	130	137	147	147	142
	GSSG	######	######	######	######	######	######	######	######	######	######
	Homocystein	101	116	127	130	126	109	120	130	137	147
	Homoserine	78	90	102	111	119	86	104	125	139	131
	S-Adenosylho	83	49	22	18	17	60	51	39	51	37
	S-Adenosylme	80	76	65	62	51	98	105	112	113	120
	Cystathionine	104	127	147	162	199	123	142	165	175	183
	Cysteamine	99	1442	7813	9363	25473	######	######	######	######	######
	Taurine	88	42	25	18	20	102	107	119	124	141
	CoA	129	151	161	171	176	150	162	178	184	179
	Acetylglycine	97	110	76	69	149	######	######	######	######	######
	Choline	1534	2497	2740	3056	2884	1701	3194	3591	3179	3089
	Betaine	4109	6346	7198	7482	6486	4390	6847	6939	6219	6205
	Aminobutyra	2690	4145	4569	5178	4765	3047	4378	4869	4670	4670
	GABA	76	85	95	109	127	105	132	218	233	297
	Hydroxybuty	102	110	91	109	104	139	148	160	159	175
	Hydroxyproli	126	167	213	216	253	138	180	212	218	222
	Phenylpyruva	89	73	41	35	12	105	109	118	127	131
	Creatine	113	122	120	120	113	131	147	159	167	162
	Creatinine	101	79	28	24	13	119	104	89	110	115
	Carnitine	######	######	######	######	######	######	######	######	######	######
	Spermidine	98	96	87	83	81	107	110	120	122	123
	3-Methyl-2-o	102	115	119	119	120	105	110	112	111	108
	3-Methyl-2-o	######	######	######	######	######	######	######	######	######	######
	4-Methyl-2-o	55	53	43	43	34	88	87	86	87	83
	5-Hydroxytry	267	824	1360	1433	1663	256	605	859	779	883
	3-Hydroxykyn	503	320	364	294	239	250	231	184	151	137
	Kynurenine	######	######	######	######	######	######	######	######	######	######
	N-Formylkynu	73	80	86	86	80	87	92	94	91	105
	Quinolinic Ac	81	79	68	71	54	93	101	111	117	117
	Serotonine (5	112	143	204	228	365	127	149	176	183	200
Urea Cycle	Carbamoylph	53	83	115	125	167	57	74	91	89	96
	Citrulline	122	121	143	147	142	112	128	147	133	129
	ArgSucc	96	92	69	62	24	94	90	84	88	89
	Ornithine	99	111	126	127	129	106	114	118	116	119
	N-Acetylornit	81	81	105	129	125	62	101	126	157	154
TCA Cycle	Citrate	174	295	558	637	1100	202	343	475	456	516
	Aconitate	773	1505	2930	3202	5354	795	1326	1826	1531	2128
	a-Ketoglutara	143	132	136	148	168	121	123	130	134	153
	Succinate	106	124	135	143	154	115	132	141	149	144
	Fumarate	223	186	219	258	306	174	178	222	233	268
	Malate	114	130	143	151	164	120	132	140	144	143
Carbohydrate Metabolites	Hexosephosp	937	678	724	739	760	450	410	365	314	307
	Fructose-1,6-	435	156	87	52	44	99	154	125	47	37
	Dihydroxyace	85	102	106	108	90	109	115	120	134	116
	3-Phosphogly	######	######	######	######	######	######	######	######	######	######
	Phosphoenol	######	######	######	######	######	######	######	######	######	######
	Pyruvate	829	629	280	211	659	1458	499	557	431	794
	Lactate	104	120	139	137	158	105	112	124	127	130
	6-Phosphoglu	108	76	56	43	31	114	98	80	76	65
	Gluconate	116	151	211	284	328	213	265	393	418	484
	Pentose-5-ph	507	703	950	1275	1443	446	596	813	1055	1379
	Erythrose-4-p	520	885	1366	1545	2087	403	626	865	951	1037
	Deoxyribose-	171	285	337	338	344	163	187	219	234	255
	Glucosamine	180	190	194	214	185	182	209	228	236	238
	Glucosamine-	######	######	######	######	######	######	######	######	######	######
	Glycerol-3-ph	98436	######	######	######	######	3499	3389	2957	7622	9045
	N-Acetylneura	1039	1894	2380	3691	4632	998	1140	1007	1214	2585
Lipids	Acetoacetate	106	118	125	128	128	123	134	144	146	143
	Mevalonate	######	######	######	######	######	######	######	######	######	######
	Mevalonate-5	108	125	147	163	186	99	111	133	163	173
	Geranylgeran	82	62	39	35	31	113	99	92	93	85
	Malonate	######	######	######	######	######	######	######	######	######	######

Lowest Value — Highest Value

Cells

Class	Compound	24h Met(+)	48h Met(+)	72h Met(+)	96h Met(+)	120h Met(+)	24h Met(-)	48h Met(-)	72h Met(-)	96h Met(-)	120h Met(-)
Amino Acids	Alanine	66	84	88	98	100	56	43	34	29	27
	Arginine	80	93	92	87	91	83	94	89	98	100
	Asparagine	80	98	85	94	100	74	43	33	39	35
	Aspartate	100	78	57	77	69	89	59	49	56	40
	Cysteine	77	81	87	91	100	82	63	72	85	80
	Glutamate	95	85	99	97	100	75	68	62	62	47
	Glutamine	47	70	81	93	100	59	56	73	84	81
	Glycine	87	93	83	95	100	87	68	53	52	44
	Proline	96	100	79	91	90	94	61	52	55	41
	Serine	66	62	81	100	92	58	44	44	56	54
	Methionine	78	98	91	93	100	16	19	20	19	21
	Histidine	75	100	85	97	97	80	70	53	63	55
	Isoleucine	79	100	81	85	80	84	81	69	80	76
	Leucine	70	100	87	95	91	84	82	69	80	70
	Lysine	63	70	78	73	72	66	79	88	96	100
	Phenylalanine	75	100	91	100	100	82	80	65	74	67
	Threonine	80	93	89	95	100	73	67	54	64	64
	Tryptophan	66	100	79	90	90	74	70	55	63	56
	Tyrosine	83	100	86	89	88	90	92	81	91	93
	Valine	85	99	92	99	100	89	84	76	86	82
Amino Acid Derivatives	Cystine	60	35	16	70	55	46	41	26	100	62
	GSH	59	100	27	70	56	50	57	73	65	55
	GSSG	16	27	33	62	100	20	17	18	17	11
	Homocystein	61	79	74	82	100	58	29	20	30	27
	Homoserine	62	100	70	94	97	91	91	71	96	77
	S-Adenosylho	62	100	68	84	76	72	80	57	70	64
	S-Adenosylme	82	100	85	87	87	88	92	80	90	92
	Cystathionine	100	71	40	49	47	74	60	48	51	39
	Cysteamine	100	77	75	85	59	79	36	37	32	30
	Taurine	100	75	54	58	53	79	65	60	53	40
	CoA	92	100	80	89	81	97	90	81	92	83
	Acetylglycine	100	46	53	83	67	96	56	43	25	18
	Choline	72	86	85	100	86	73	73	63	67	63
	Betaine	69	100	78	97	70	69	62	41	47	42
	Aminobutyra	46	82	76	100	76	56	55	41	49	44
	GABA	63	100	77	94	88	70	79	55	61	62
	Hydroxybuty	80	100	92	93	91	67	70	73	74	70
	Hydroxyproli	94	84	100	96	100	74	66	60	60	45
	Phenylpyruva	85	100	90	100	96	86	82	63	67	58
	Creatine	52	68	68	100	96	66	66	52	53	40
	Creatinine	35	30	26	29	28	53	95	96	100	90
	Carnitine	77	100	63	92	75	69	57	37	59	23
	Spermidine	95	94	90	100	98	92	78	66	57	39
	3-Methyl-2-o	87	89	77	100	84	83	68	59	78	54
	3-Methyl-2-o	61	32	75	51	30	54	44	100	74	54
	4-Methyl-2-o	50	45	59	69	100	50	54	76	72	84
	5-Hydroxytry	100	79	69	76	71	89	64	53	46	52
	3-Hydroxykyn	69	60	37	38	26	100	50	47	49	47
	Kynurenine	61	58	25	34	17	100	46	38	55	31
	N-Formylkynu	67	100	87	83	72	55	47	51	50	56
	Quinolinic Ac	80	93	89	95	100	73	67	54	64	64
	Serotonine (5	62	100	86	73	66	73	85	79	76	76
Urea Cycle	Carbamoylph	49	80	72	100	70	64	45	18	32	29
	Citrulline	52	60	100	67	56	51	60	82	74	81
	ArgSucc	76	78	79	75	69	78	79	93	98	100
	Ornithine	72	100	72	79	90	79	70	58	74	66
	N-Acetylornit	86	100	92	93	91	91	92	83	91	84
TCA Cycle	Citrate	51	100	45	59	54	52	48	31	38	31
	Aconitate	53	60	77	100	94	40	32	22	32	21
	a-Ketoglutara	76	100	95	96	89	71	98	85	91	82
	Succinate	60	93	77	100	60	67	56	34	54	52
	Fumarate	57	66	58	69	45	42	54	37	50	39
	Malate	65	100	61	77	80	70	69	54	59	40
Carbohydrate Metabolites	Hexosephosp	82	97	100	96	69	86	82	81	93	65
	Fructose-1,6-	58	51	100	79	67	60	51	73	84	74
	Dihydroxyace	89	100	98	82	58	62	68	36	72	83
	3-Phosphogly	45	56	75	62	33	41	40	39	83	100
	Phosphoenol	62	59	53	42	92	65	98	84	100	82
	Pyruvate	56	43	100	71	44	52	42	58	86	82
	Lactate	100	87	55	55	61	64	49	60	64	53
	6-Phosphoglu	49	99	47	43	30	63	84	63	100	96
	Gluconate	54	91	87	100	84	64	68	47	70	57
	Pentose-5-ph	37	45	32	36	30	37	68	94	100	76
	Erythrose-4-p	59	65	60	65	57	52	79	86	100	90
	Deoxyribose-	73	100	75	93	73	76	85	71	74	64
	Glucosamine	65	72	62	58	83	66	61	78	100	78
	Glucosamine-	90	85	87	94	83	86	100	86	95	100
	Glycerol-3-ph	64	62	100	85	58	71	64	62	80	60
	N-Acetylneura	95	97	70	92	75	100	83	70	73	65
Lipids	Acetoacetate	65	77	66	65	61	61	81	85	100	97
	Mevalonate	######	######	######	######	######	######	######	######	######	######
	Mevalonate-5	92	100	99	97	94	94	95	95	85	78
	Geranylgeran	27	64	74	92	100	44	35	49	66	57
	Malonate	######	######	######	######	######	######	######	######	######	######

Lowest Value — Highest Value

Media

Class	Compound	24h Met(+)	48h Met(+)	72h Met(+)	96h Met(+)	120h Met(+)	24h Met(-)	48h Met(-)	72h Met(-)	96h Met(-)	120h Met(-)
Pyrimidines	Carbamoylasp	181	324	668	603	938	161	241	249	243	219
	Dihydroorota	######	######	######	######	######	######	######	######	######	######
	Orotate	######	######	######	######	######	######	######	######	######	######
	Uracil	######	######	######	######	######	######	######	######	######	######
	Uridin	2	3	14	0	1	2	3	5	1	7
	UMP	231	220	200	200	184	241	232	221	211	196
	UDP	######	######	######	######	######	######	######	######	######	######
	UTP	######	######	######	######	######	######	######	######	######	######
	dUridine	######	######	######	######	######	######	######	######	######	######
	dUMP	######	######	######	######	######	######	######	######	######	######
	dUDP	112	134	161	177	198	137	161	182	197	189
	dUTP	######	######	######	######	######	######	######	######	######	######
	UDP-Glc	98	102	97	100	82	115	126	130	136	136
	UDP-Glucuro	######	######	######	######	######	######	######	######	######	######
	Cytosin	######	######	######	######	######	######	######	######	######	######
	Cytidin	######	######	######	######	######	######	######	######	######	######
	CMP	4068	10843	7356	4366	4530	1807	1433	933	493	1018
	CDP	147	122	114	125	107	128	144	153	165	171
	CTP	######	######	######	######	######	######	######	######	######	######
	dCytidine	######	######	######	######	######	######	######	######	######	######
	dCMP	######	######	######	######	######	######	######	######	######	######
	dCDP	293	190	166	178	198	309	194	121	123	204
	dCTP	94	108	115	121	124	111	123	132	133	135
	Thymin	223	211	148	111	103	239	166	116	106	112
	Thymidine	######	######	######	######	######	######	######	######	######	######
	TMP	######	######	######	######	######	######	######	######	######	######
	TDP	######	######	######	######	######	######	######	######	######	######
	TTP	######	######	######	######	######	######	######	######	######	######
	dThymidine	######	######	######	######	######	######	######	######	######	######
	dTMP	36	44	100	42	42	42	42	53	43	48
	dTDP	1	8	109	0	0	4	3	11	0	5
	dTTP	######	######	######	######	######	######	######	######	######	######
Purines	Inosin	462	514	538	599	871	176	141	147	299	1776
	dInosin	244	429	583	451	540	166	362	402	519	754
	IMP	######	######	######	######	######	######	######	######	######	######
	Adenin	1827	1149	1443	1784	1574	989	817	846	1085	1440
	Adenosin	######	######	######	######	######	######	######	######	######	######
	AMP	######	######	######	######	######	######	######	######	######	######
	ADP	0	0	0	0	0	######	######	######	######	######
	ATP	######	######	######	######	######	######	######	######	######	######
	cAMP	######	######	######	######	######	######	######	######	######	######
	dAMP	######	######	######	######	######	######	######	######	######	######
	dADP	######	######	######	######	######	######	######	######	######	######
	dATP	######	######	######	######	######	######	######	######	######	######
	Guanin	######	######	######	######	######	######	######	######	######	######
	Guanosin	335	11444	78	34	427	39	111	489	594	1010
	GMP	168	118	131	97	95	102	119	118	112	134
	GDP	3	2	2	2	2	4	4	16	18	30
	GTP	######	######	######	######	######	######	######	######	######	######
	Hypoxanthin	147	463	297	275	514	84	204	433	395	1299
	Xanthin	128	155	170	183	199	147	168	205	225	267
	Xanthosin	87	75	81	57	72	91	72	65	38	32
	Xanthurenic A	######	######	######	######	######	######	######	######	######	######
	XMP	######	######	######	######	######	######	######	######	######	######
Co-Factors	Riboflavine	######	######	######	######	######	######	######	######	######	######
	FMN	######	######	######	######	######	######	######	######	######	######
	FMNH2	######	######	######	######	######	######	######	######	######	######
	FAD	######	######	######	######	######	######	######	######	######	######
	FADH2	######	######	######	######	######	######	######	######	######	######
	Nicotinamide	######	######	######	######	######	######	######	######	######	######
	Nicotinate	######	######	######	######	######	######	######	######	######	######
	NAD+	######	######	######	######	######	######	######	######	######	######
	NADH	######	######	######	######	######	######	######	######	######	######
	NADP+	######	######	######	######	######	######	######	######	######	######
	NADPH	87	85	76	75	63	92	95	97	99	98
	Dihydrofolate	113	117	119	123	123	125	137	152	162	194
	Tetrahydrofol	105	113	120	126	128	121	136	149	154	153
	5-Methyl-THF	112	106	79	73	37	129	123	122	132	123
	Tetrahydrobi	57	52	68	67	64	78	69	94	78	62
	Thiamine (+)	######	######	######	######	######	######	######	######	######	######
	Thiamine pho	######	######	######	######	######	######	######	######	######	######
	Pantothenate	116	128	148	146	144	140	150	166	145	134
	Pyridoxine	######	######	######	######	######	######	######	######	######	######

Lowest Value — Highest Value

Cells

Class	Compound	24h Met(+)	48h Met(+)	72h Met(+)	96h Met(+)	120h Met(+)	24h Met(-)	48h Met(-)	72h Met(-)	96h Met(-)	120h Met(-)
Pyrimidines	Carbamoylasp	49	43	74	100	14	8	54	7	52	3
	Dihydroorota	91	48	31	60	35	100	40	34	40	30
	Orotate	100	84	60	87	74	98	97	73	77	46
	Uracil	92	37	11	29	15	100	10	9	14	42
	Uridin	99	77	58	79	54	100	78	54	70	43
	UMP	100	98	96	90	91	89	91	91	90	85
	UDP	######	######	######	######	######	######	######	######	######	######
	UTP	59	43	71	62	100	52	44	89	63	52
	dUridine	52	100	32	40	15	62	78	55	69	74
	dUMP	100	53	24	49	22	18	19	26	36	31
	dUDP	76	100	84	88	88	82	93	81	94	87
	dUTP	######	######	######	######	######	######	######	######	######	######
	UDP-Glc	53	77	35	17	12	58	84	69	100	92
	UDP-Glucuro	100	81	48	49	18	65	19	7	11	7
	Cytosin	######	######	######	######	######	######	######	######	######	######
	Cytidin	######	######	######	######	######	######	######	######	######	######
	CMP	67	95	78	100	86	71	88	69	69	65
	CDP	64	84	54	64	75	67	66	72	100	75
	CTP	58	55	27	0	100	58	0	0	0	95
	dCytidine	24	27	21	51	81	45	100	17	11	12
	dCMP	######	######	######	######	######	######	######	######	######	######
	dCDP	100	75	66	81	78	67	64	76	69	86
	dCTP	100	82	85	86	91	75	69	75	79	81
	Thymin	88	100	73	79	81	81	95	82	97	93
	Thymidine	50	77	84	64	0	50	42	100	0	36
	TMP	100	51	46	61	56	98	49	62	61	55
	TDP	57	53	91	100	65	47	33	34	31	16
	TTP	30	28	31	33	61	32	60	99	99	100
	dThymidine	88	81	68	100	68	91	84	59	71	53
	dTMP	94	93	98	100	96	76	89	63	41	20
	dTDP	96	92	98	100	95	76	89	64	41	18
	dTTP	91	39	14	46	22	100	10	6	17	45
Purines	Inosin	100	57	57	80	54	87	39	36	46	36
	dInosin	99	43	49	89	70	100	55	35	65	42
	IMP	84	67	62	93	66	100	77	79	93	61
	Adenin	53	64	58	85	70	67	100	92	100	67
	Adenosin	85	62	31	63	46	100	39	29	55	55
	AMP	######	######	######	######	######	######	######	######	######	######
	ADP	100	41	29	22	10	49	0	1	4	3
	ATP	100	89	61	49	23	47	13	7	6	7
	cAMP	######	######	######	######	######	######	######	######	######	######
	dAMP	100	61	30	15	20	11	9	0	0	19
	dADP	98	63	51	67	62	100	51	62	61	60
	dATP	72	85	68	76	79	67	100	87	72	52
	Guanin	100	46	2	31	21	98	8	1	15	50
	Guanosin	100	72	61	36	18	77	57	60	16	7
	GMP	100	53	50	57	60	76	38	36	31	18
	GDP	80	80	89	89	100	81	77	87	77	61
	GTP	81	88	89	89	97	78	81	100	91	87
	Hypoxanthin	87	66	80	86	100	83	64	78	65	45
	Xanthin	68	68	76	83	100	55	72	60	58	42
	Xanthosin	74	78	91	79	100	55	71	47	43	25
	Xanthurenic A	71	56	70	74	100	68	52	55	32	29
	XMP	54	100	62	40	81	42	39	38	24	13
Co-Factors	Riboflavine	52	100	72	76	60	64	81	52	67	44
	FMN	######	######	######	######	######	######	######	######	######	######
	FMNH2	65	70	88	97	100	69	66	74	77	54
	FAD	######	######	######	######	######	######	######	######	######	######
	FADH2	93	63	95	89	92	92	79	82	100	62
	Nicotinamide	35	0	48	32	100	72	11	0	0	9
	Nicotinate	78	45	42	65	62	100	68	70	73	11
	NAD+	64	41	35	62	75	100	87	52	94	23
	NADH	97	58	73	91	92	100	86	82	75	34
	NADP+	48	57	39	87	97	71	56	25	100	30
	NADPH	77	80	81	91	100	76	90	75	76	62
	Dihydrofolate	94	72	70	68	68	71	84	75	73	70
	Tetrahydrofol	100	79	54	56	48	81	68	61	48	29
	5-Methyl-THF	45	100	32	13	10	60	58	44	68	59
	Tetrahydrobi	100	95	86	84	71	92	71	64	66	66
	Thiamine (+)	5	3	2	1	0	8	60	98	100	93
	Thiamine pho	83	10	25	3	0	0	82	50	0	100
	Pantothenate	63	84	79	100	94	48	37	36	41	37
	Pyridoxine	100	90	76	82	72	98	67	36	63	60

Lowest Value — Highest Value
